# Microchip capillary electrophoresis dairy device using fluorescence spectroscopy for detection of ciprofloxacin in milk samples

**DOI:** 10.1038/s41598-020-70566-1

**Published:** 2020-08-11

**Authors:** Rick Bosma, Jasen Devasagayam, Ashutosh Singh, Christopher M. Collier

**Affiliations:** Applied Optics and Microsystems Laboratory, University of Guelph, Guelph, ON N1G 2W1 Canada

**Keywords:** Biomedical engineering, Optical sensors, Optoelectronic devices and components, Imaging and sensing, Micro-optics

## Abstract

Detecting antibiotics in the milk supply chain is crucial to protect humans from allergic reactions, as well as preventing the build-up of antibiotic resistance. The dairy industry has controls in place at processing facilities, but controls on dairy farms are limited to manual devices. Errors in the use of these manual devices can result in severe financial harm to the farms. This illustrates an urgent need for automated methods of detecting antibiotics on a dairy farm, to prevent the shipment of milk containing antibiotics. This work introduces the microchip capillary electrophoresis dairy device, a low-cost system that utilizes microchip capillary electrophoresis as well as fluorescence spectroscopy for the detection of ciprofloxacin contained in milk. The microchip capillary electrophoresis dairy device is operated under antibiotic-absent conditions, with ciprofloxacin not present in a milk sample, and antibiotic-present conditions, with ciprofloxacin present in a milk sample. The response curve for the microchip capillary electrophoresis dairy device is found through experimental operation with varied concentrations of ciprofloxacin. The sensitivity and limit of detection are quantified for the microchip capillary electrophoresis dairy device.

## Introduction

Of critical importance is the prevention of antibiotic contamination in the milk supply chain. This prevention of antibiotic contamination protects human beings against a number of health issues including antibiotic resistance^1^ and allergic reactions^2^. Antibiotic contamination is of particular concern for the dairy industry, where antibiotics such as ß-lactams, cephalosporins, tetracyclines, and fluoroquinolones can contaminate milk due to treatment of bovine ailments^3,4^. As such, devices that are capable of detecting antibiotics in milk are of interest for the dairy industry. However, at the dairy farm level, there are only manual devices available, including prepared incubated tests such as the Delvotest T^5^ and lateral flow assays such as the Charm ROSA test^6^. These manual devices subject dairy farms to substantial human error, resulting in situations where dairy farmers will ship milk that is contaminated with antibiotics to processing plants, suffer monetary losses, and (after multiple offences) suffer loss of licensure^7^.

The Delvotest T test is a highly sensitive detection system which is able to detect a wide range of antibiotic residues, especially for tetracyclines and *β*-lactams, as it measures the inhibitory substance in the milk sample, which is the antibiotic residue at a concentration above the limit of detection (LOD) of the test^5^. Milk samples are added to an agar medium, which contains spores of *Geobacillus stearothermophilus* var*. calidolactis*, along with glucose (a fermentable sugar), and a pH colour indicator, bromocresol purple. The test is incubated for several hours. If the antibiotic residue in the sample is below the LOD, the bacillus spores will germinate, producing an acid from fermentation. Hence, antibiotic residues below the LOD will cause the indicator to change colour from purple to yellow. Antibiotic residues above the LOD inhibits the germination of the bacillus spores and fermentation does not occur so the colour of the indicator is left unchanged^5^.

The Charm Rapid One-Step Assay (ROSA) test works similarly, where a mixture of the milk sample and buffer solution is incubated within the sample compartment of the test strip^6^. It works by utilizing a lateral-flow format with gold-bead receptors which detect and measure β-lactam and tetracycline antibiotics in a milk sample. After adding the milk to the test strip, it is incubated for several minutes, which will rehydrate and mobilize the receptors. These receptors are carefully designed to have a sensitivity which will detect antibiotics at concentrations below the maximum residual limit (MRL). Receptors which are unreacted with the antibiotic will bind to form a red test line. Receptors which react with the antibiotics pass across this test line and bind with the control line of the test strip, causing the control line to darken. Hence, if the colour of the indicator line is lighter than the control line on the strip, or even if the line is not formed, it signifies the presence of specific antibiotics in milk^8^. The Delvotest T and Charm ROSA tests are both manual processes which would require well-trained personnel to make qualitative decisions.

To reduce the manual error associated with manual devices (as discussed above), laboratory device tests may be employed to detect the presence of antibiotics in milk. These laboratory device tests include mass spectrometry^9^, liquid chromatography^10^, immunoassay^11^, potentiometric^12^, and surface plasmon resonance^13^ devices. However, these laboratory devices are slow because they require cumbersome processes including transportation off-site from the dairy farm, sample preparation, and extensive incubation periods^9,12^. The temporal limitations of laboratory devices are in stark contrast to the demands of on-farm antibiotic device detection, where full antibiotic detection should ideally occur within a few minutes during the milking process for each individual cow. It should be considered that if antibiotic detection takes place on the combined milk repository from many cows, the entire milk repository is in jeopardy if any antibiotic contamination occurs. Additionally, it is important to be able to develop a system that could potentially be used by untrained personnel, which microfluidic devices can offer.

This work responds to the need for on-site detection of antibiotics, such as ciprofloxacin, in milk samples through the development of a microchip capillary electrophoresis dairy device^14^. Actuation and sensing aspects of microfluidic technology^15^ are integrated, as lab-on-a-chip microfluidic technology is a commonly used method for sensing purposes^14,15^. The microchip capillary electrophoresis dairy device combines respective microchip capillary electrophoresis and fluorescence spectroscopy^16–18^ techniques. The microchip capillary electrophoresis technique leverages capillary electrophoresis, whereby a controlled electric field is strategically used to separate constituent components and isolate the analyte(s) within a microfluidic channel^19^. The fluorescence spectroscopy technique is utilized to achieve the ultimate detection of an important antibiotic, ciprofloxacin. Ciprofloxacin is an ideal choice of antibiotic for this study because it is a metabolite of the commonly used antibiotic, enrofloxacin, which is currently approved for widespread use in regions of the world and there are strict regulations on the maximum residual levels of the sum of enrofloxacin and ciprofloxacin^20,21^. To introduce and develop the microchip capillary electrophoresis dairy device, this work discusses the design and fabrication of the device, chemicals used, operation, antibiotic-absent and antibiotic-present operation, and ciprofloxacin detection.

It should be noted that there are several relevant studies to our work that involve combinations of capillary electrophoresis and antibiotic study^22–34^. These studies are primarily larger channels while our presented work focuses on smaller channels and considerably shorter channel lengths. Many of these mentioned studies work with effective lengths ranging from 500 to 960 mm with inner capillary diameters of 50–75 μm. For example, in a study by Springer et al., the capillary used had an effective length of 525 mm and an inner diameter of 75 μm, while applying up to 20 kV to create the electric field^26^. In another example, Lara et al. used a capillary with an effective length of 960 mm and an inner diameter of 50 μm, while applying up to 25 kV to the electrodes^24^. In comparison, our microchip capillary system utilizes microcapillaries possessing an effective length of 79 mm with an inner diameter of 50 μm. This makes it possible to use lower voltages to achieve equivalent electric field strengths, while also achieving faster fluid velocities as a result of the smaller channel widths. This microscale scalability is particularly important in highly-parallel applications requiring high-throughput analyses, such as is required on dairy farms^27^. Another factor to consider is the sample preparation required, where the aforementioned studies require solid-phase extraction of milk samples using centrifugation to elute the antibiotic residues. In comparison, the presented microchip capillary electrophoresis dairy device only requires filtering before being injected into the device, as the pinched sample injection will send a minute sample for detection. Milk-based, scalable, and accessible systems, as in the presented work, are of great interest to the large dairy research community.

## Results

### Fluorescence spectroscopy for detection

To allow for future potential of mass-market adoption of the microchip capillary electrophoresis dairy device, cost minimization is an important design factor that is considered. Therefore, the fluorescence spectroscopy technique is implemented with low-cost components, being an ultraviolet (UV) light emitting diode (LED) for the fluorescence excitation and a photodiode for the fluorescence detection, as in previous work^35^. As ciprofloxacin emits fluorescence at an excitation wavelength of 270 nm^36^, the UV LED for fluorescence excitation (VLMU60CL00-280–125 Vishay USA) is selected for its wavelength range of 270–290 nm and sufficient optical power of 2.4 mW. The UV LED is fixed on the UV LED printed circuit board (PCB). As ciprofloxacin emits fluorescence at a wavelength of 440 nm^36^, an optical bandpass filter (86-339, Edmund Optics, USA) is selected for its passband wavelength range of 420–460 nm. There was an initial attempt to use an optical long pass filter (FGL435, Thorlabs USA), however, this optical long pass filter suffered from severe self-fluorescence. The light passed through the optical bandpass filter is then detected by a photodiode mounted on the detection PCB. While commercially available photodiodes with peak response at wavelength of 440 nm are unavailable, the SFH 2440 photodiode manufactured by Osram (Munich, Germany) has a spectral sensitivity of 40% of its maximum at this wavelength of 440 nm, which is sufficient. The photocurrent generated from the photodiode is amplified by a transimpedance circuit with a gain of 10^9^. The output signal from the transimpedance circuit is passed through an electrical lowpass filter and the resulting signal is recorded by a National Instruments USB-6341 data acquisition system (controlled by a computer).

### Fluid actuation by electric field application

To apply electric potential to the microfluidic chip, platinum electrodes (Surepure Chemetals, USA) are chosen, as platinum will not corrode under the experimental conditions of the microchip capillary electrophoresis dairy device. Platinum electrodes were formed from 1.295 mm diameter platinum wire with tapered ends to allow insertion into microfluidic wells without contacting the sides of the wells. Retention plates help secure the platinum electrodes in place. Copper wire electrodes, being a low-cost alternative, were initially used, however, severe corrosion occurred.

The voltage application to the microfluidic chip was regulated by a four channel, laboratory-built voltage sequencer with each channel capable of supplying 0–500 V. This voltage sequencer was energized by a Keithley 2290-5 power supply.

### Operational procedures

**Figure 1:**
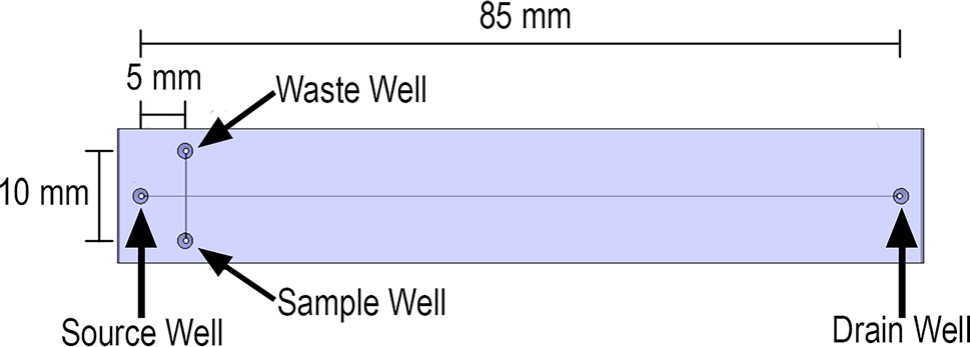
Diagram of the T8050 Glass Microchip with a double-T junction at the separation channel.

Because the developed system requires complicated design and fabrication of multiple components, a commercially available microfluidic chip with proven functionality is chosen, being the T8050 microfluidic chip from Micronit (Enschede, Netherlands). The name of each well is illustrated in the schematic of the microfluidic chip is presented in Fig. 1.

Prior to experimentation, it is necessary to power the ultraviolet (UV) light emitting diode (LED) for 90 min to allow the output of the microchip capillary electrophoresis dairy device to stabilize. (This UV LED could be replaced to reduce this cumbersome warmup period.) In addition, the 1 M NaOH solution is injected into the microfluidic chip under pressure from a syringe (3 mL) until all four wells are filled. The NaOH is left in the microfluidic chip for 15 min and then washed out with Millipore water. The microfluidic chip is then drained and filled with citrate buffer. The citrate is emptied from the sample well using a pipette and the well is subsequently filled with filtered milk, for antibiotic-absent operation, or a solution of milk and ciprofloxacin hydrochloride monohydrate, for antibiotic-present operation. It is envisioned that a future iteration of this system would incorporate automated chip preparation and sample loading in order to create a fully automated microchip capillary electrophoresis dairy device. This would make it feasible to implement the system on dairy farms for farmers to reliably use.

Flow of the buffer solution through the microfluidic channel is a result of electroosmotic flow and segregation of the milk sample into its composing elements within the electroosmotic flow is a result of electrophoresis. These combined effects (electroosmotic flow and electrophoresis) on the velocity of any given element within the buffer solution is

1 $$u = { }u_{EOF} + u_{EP} = { }\left( {\mu_{EOF} + { }\mu_{EP} } \right)E.$$

Here the velocity of an element is *u*, the velocity from electroosmotic flow is $u_{EOF}$, the electroosmotic flow mobility is $\mu_{EOF}$, the velocity induced by electrophoresis is $u_{EP}$, the electrophoresis mobility is $\mu_{EP}$, and the applied electric field is $E$. The electroosmotic flow mobility is typically much larger compared to the electrophoresis mobility. As such, the electroosmotic flow velocity determines the net direction of the elements and the electrophoresis velocity determines how elements separate in the net flow.

**Figure 2:**
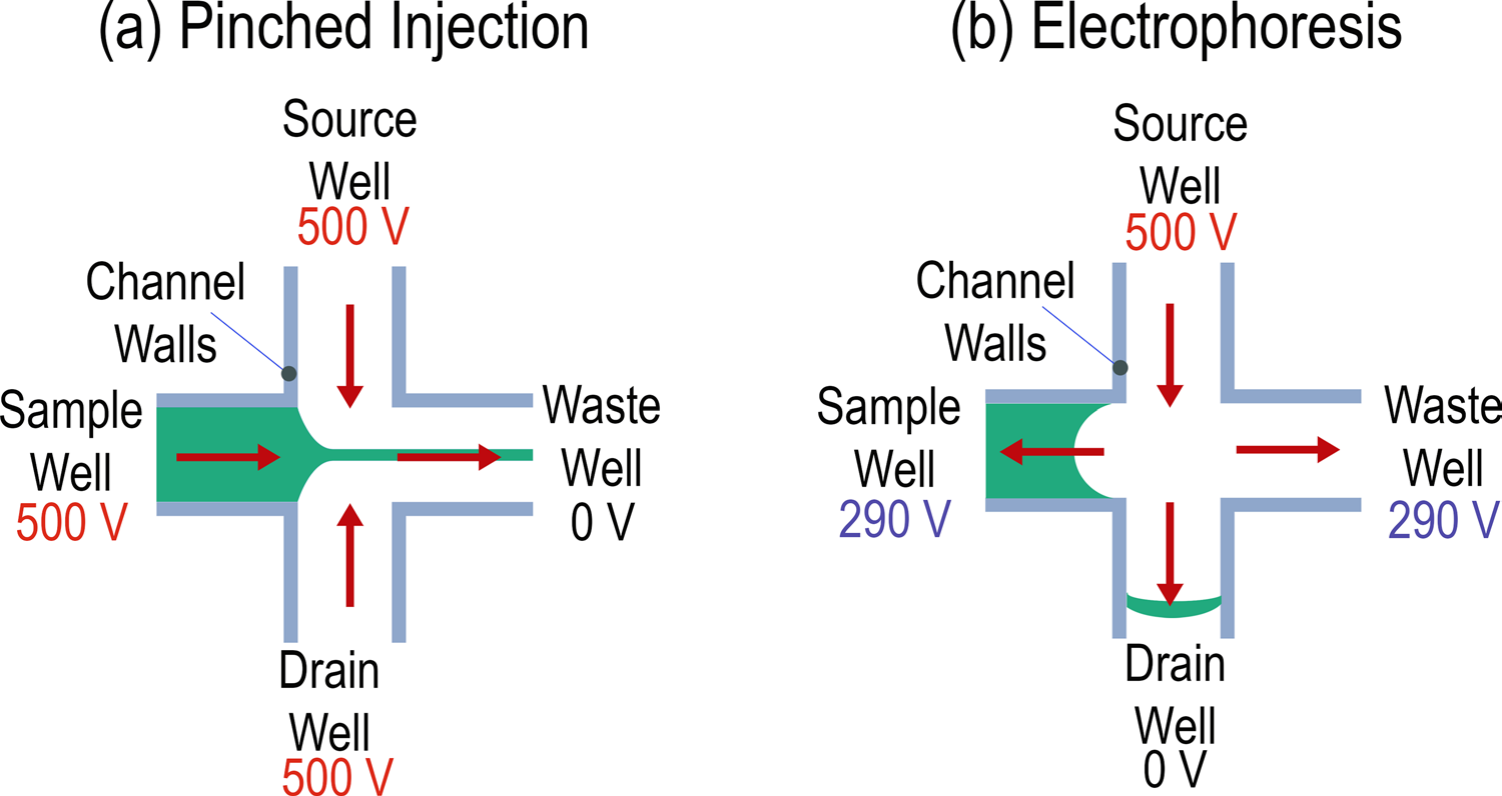
(**a**) Electric field configuration for pinched sample injection. This portion of the experiment fills the injection channel. (**b**) Electric field configuration for separation. This portion of the experiment takes a pinched sample from the injection step and sends this small sliver towards the drain well.

During operation of the microchip capillary electrophoresis dairy device, application of 500 V to the sample, source, and drain wells results in the sample being pulled towards the waste well. The waste well is at a potential of 0 V. This is pinched sample injection, as illustrated in Fig. 2a, with a high potential applied to the sample, source, and drain wells and ground potential applied to the waste well. The result is electroosmotic flow from sample, source, and drain wells to the waste well. To perform the electrophoresis, 500 V is applied to the source well, 290 V is applied to the sample and waste wells, and 0 V is applied to the drain well, as in Fig. 2b. The visualization of this flow would reveal the sample is pinched by the flows from the source and drain while entering the waste channel, as in Fig. 2a, and then a narrow plug travelling towards the drain well, as in Fig. 2b.

### Antibiotic-absent and antibiotic-present operation

**Figure 3:**
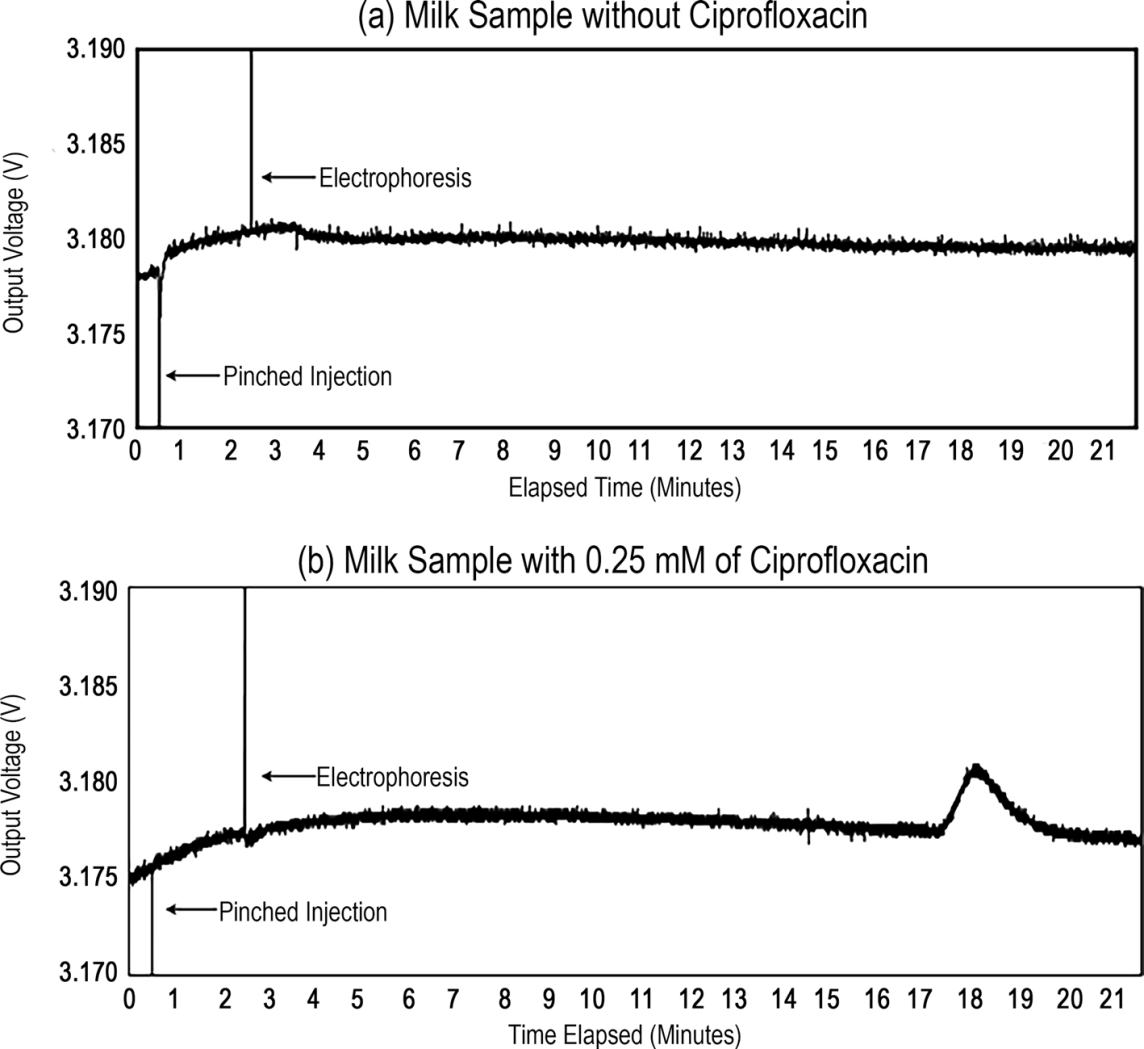
(**a**) Antibiotic-absent test of detection system by testing with milk without ciprofloxacin. (**b**) Antibiotic-present test of detection system by testing with milk with 0.25 mM concentration of ciprofloxacin.

The microchip capillary electrophoresis dairy device is first operated with a milk sample containing no antibiotics to establish an antibiotic-absent response. This antibiotic-absent response is presented in Fig. 3a. When the injection begins, three of the four platinum electrodes rapidly transition potential from 0 to 500 V. This results in a noise spike in the recorded data and allows observation of the onset of sample loading and electrophoresis. This is noted in Fig. 3a with injection starting at 30 s and electrophoresis starting at two minutes and 30 s. It should be observed that as the sample approaches the UV LED for measurement at approximately 18 min, there is no discernable response, indicating the ciprofloxacin is not present. It should be noted that through careful selection of voltages and channel lengths (with the electric field being approximately equal to the ratio between these values), it is envisioned that the analysis time can be greatly reduced, in accordance with dairy requirements for analysis time^37^.

The microchip capillary electrophoresis dairy device is then operated with a milk sample containing 0.25 mM ciprofloxacin to establish an antibiotic-present response (being the change of the output voltage at the temporal region of interest). This antibiotic-present response is presented in Fig. 3b. As previously noted, the noise spikes associated with sample loading and electrophoresis are observed at 30 s and two minutes and 30 s, respectively. As the sample approaches the UV LED for measurement, at the 18 min temporal region of interest, there is a clear electrophoretic peak, indicating the presence of ciprofloxacin in the milk sample.

The Fig. 3 results should be discussed in terms of potential comigration, where another analyte may travel along with the ciprofloxacin and interfere with the measured signal. The antibiotic-absent experiment of Fig. 3a is performed without ciprofloxacin in milk, represented by a flat response. The antibiotic-absent experiment of Fig. 3b is performed with antibiotics in milk, and this is the only change from the first experiment. Since only the ciprofloxacin was added, the electrophoretic peak shown in Fig. 3b only represents the fluorescence emitted by the (isolated) ciprofloxacin. As such, comigration did not play a role.

### Operation with varied ciprofloxacin concentrations

**Figure 4:**
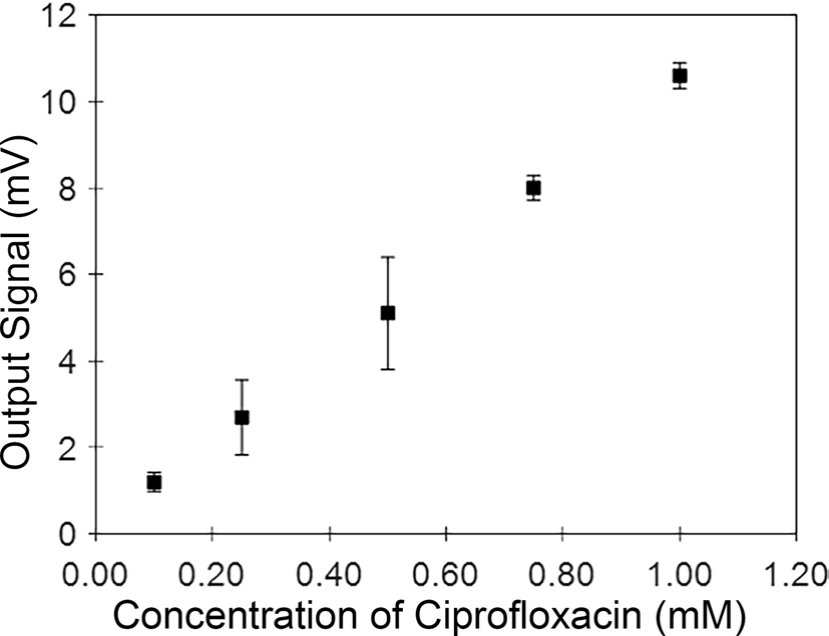
The change in the output voltage as it responds to various concentrations of ciprofloxacin in milk is shown. Each concentration is repeated (three trials) with the mean displayed. The linear fit *R*^2^ value is 0.99. Error bars are standard error, being the standard deviation divided by the number of trials.

To determine the response curve of the microchip capillary electrophoresis dairy device (being the change of the output voltage versus ciprofloxacin concentration), multiple concentrations of ciprofloxacin are injected (in separate experimental runs) and the corresponding output voltages are measured. The results are as shown in Fig. 4. It can be seen that the change in the output voltage from the microchip capillary electrophoresis dairy device increases with increasing ciprofloxacin concentration. It should be mentioned that at concentrations lower than 0.1 mM, the output signal could not be distinguished from the system noise.

To determine the sensitivity of the microchip capillary electrophoresis dairy device, the slope of the response curve is inspected. The sensitivity is found to be 10.5 mV/mM. To determine the limit of detection of the microchip capillary electrophoresis dairy device, data is collected over 10 min with a blank sample. Under these blank sample conditions, the limit of detection is found to be 0.19 mM. The method for determining the limit of detection is reported in the literature^25^. Here, the limit of detection is calculated as three times the standard deviation (for 99% certainty) of the noise within the system and comparing to the peak height for a known concentration.

At high concentrations (i.e., beyond the 1 mM upper domain of Fig. 4), the ciprofloxacin does not stay in solution. This results in the microfluidic channels (within the microfluidic chip) becoming clogged. We determine experimentally that when mixing ciprofloxacin and milk at concentrations of 1 mM and greater, the ciprofloxacin no longer dissolves in the milk, meaning that it becomes saturated in the solution. At a concentration of 1 mM, the solution was at the onset of no longer being homogeneous. As noted in the literature^38^, the maximum concentration of ciprofloxacin in cow milk is 0.09 mM (30 mg/L). Therefore, it is unlikely that the clogging observed at higher concentrations would be observed for typical on-farm applications of the microchip capillary electrophoresis dairy device. The milk samples used in this study had ciprofloxacin manually added and mixed. As such, these lab samples were made at ciprofloxacin concentrations higher than those found in nature, to demonstrate proof-of-concept. Future work can be focused on further improvement to the MCE device to reduce the limit of detection for exact industry dairy applications.

Operation of the microchip capillary electrophoresis dairy device as a sensor, with battery operation, can be discussed. Although it is envisioned that in a dairy barn environment, the device would be permanently installed in milking parlours (with main power available), the low power draw of the electronics creating the high voltage (from associated low current) may enable battery operation. This would require an integrated boost convertor and can be considered in the final market implementation of the microchip capillary electrophoresis dairy device.

## Conclusions

The microchip capillary electrophoresis dairy device developed has been shown to be capable of distinguishing between samples of milk without ciprofloxacin and samples of milk with ciprofloxacin, with a linear correlation between concentrations of ciprofloxacin and output voltage. Additionally, operation parameters (i.e., the working voltages and system warmup time) have been established. The microchip capillary electrophoresis dairy device was found to have a limit of detection of 0.19 mM of ciprofloxacin and sensitivity of 10.5 mV/mM. Future work could be focused on lowering the limit-of-detection and the analysis time, for use in regulatory control.

## Methods

### Materials and reagents

Ciprofloxacin hydrochloride monohydrate is purchased from Fischer Scientific (Ottawa, Canada). Sodium hydroxide (NaOH), sodium citrate dihydrate, and citric acid are purchased from Sigma Aldrich (Oakville, Canada). Two percent milk is purchased from a local vendor. Water used in this study is purified by a Milli-Q water purification system (Millipore, USA) to form Milli-Q water.

### Solution preparation

Milli-Q water is used to make 1 M NaOH solution and 0.1 M citrate buffer solution. The buffer solution pH is adjusted to a pH of 5.5 using NaOH. The two percent milk is filtered through 0.45 µM syringe filters prior to use. Ciprofloxacin hydrochloride monohydrate is added to two percent milk to make solution at concentrations of 1.00, 0.75, 0.50, 0.25, and 0.10 mM.

### Fixture fabrication

In the microchip capillary electrophoresis dairy device, the UV LED, fluorescence detection PCB, platinum electrodes, and the microfluidic chip all need to be accurately located relative to each other, while blocking ambient light and preventing short circuits. Therefore, top and base plates are designed from opaque acetal resin and machined to accommodate proper alignment. The top plate positions the UV LED and platinum electrodes, which is located relative to the base plate using datum alignment pins. The bottom plate positions the microfluidic chip and the fluorescence detection PCB (containing the photodiode). This design allows for the removal of the top plate, along with the platinum electrodes and circuit boards while the sample is pipetted into the microfluidic chip.

The top and base plates to hold the microfluidic chip are designed using SolidWorks and machined from opaque acetal resin (DuPont, USA) using a Tormach 770 PCNC (Tormach, USA). The UV LED and fluorescence PCBs are laboratory designed using Altium Designer (Altium, USA) and manufactured by PCB Unlimited (OR, USA).

**Figure 5:**
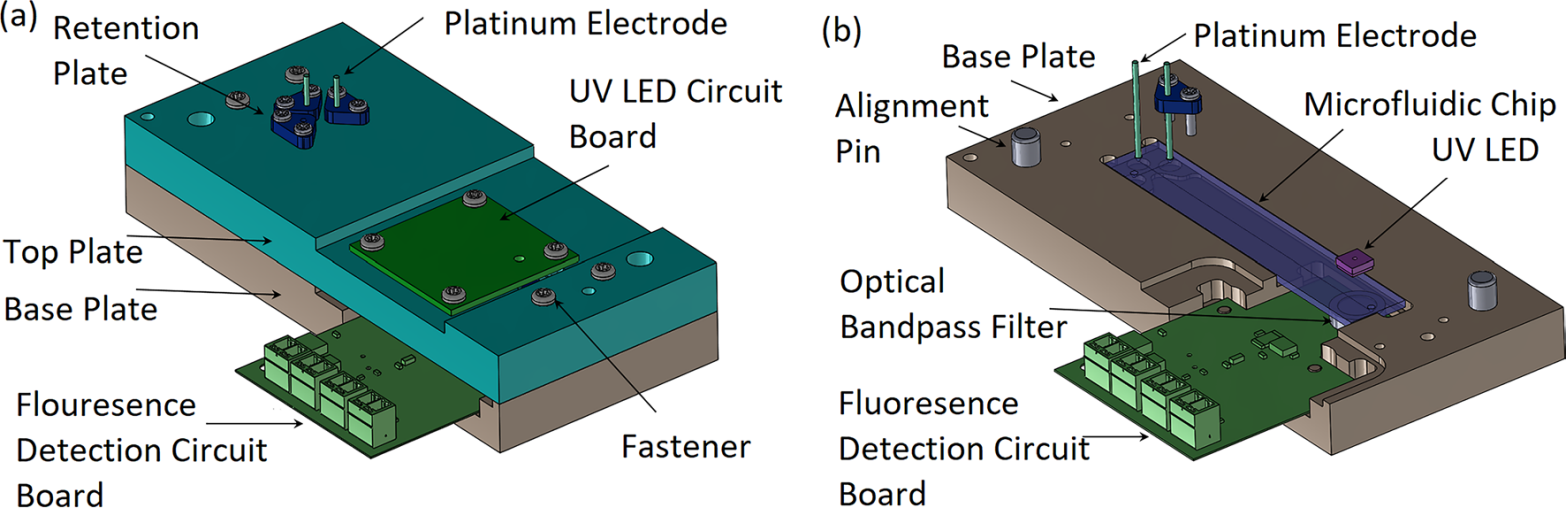
(**a**) Complete microchip capillary electrophoresis dairy device. Enclosed in the fixture are the glass microchip, detection electronics, with the platinum electrodes held by the retention plates. (**b**) Complete microchip capillary electrophoresis dairy device with the top plate removed. Seen here is the microfluidic chip with the UV LED above the chip for analyte excitation.

The complete microchip capillary electrophoresis dairy device is presented in Fig. 5a, and the microchip capillary electrophoresis dairy device with the top plate removed is presented in Fig. 5b (to reveal the based plate, microfluidic chip, and optical components).
